# Natural Reservoir of *Trypanosoma cruzi* Found in Triatomines Targeting Humans: Results from Nation-wide Vector Surveillance in El Salvador

**DOI:** 10.31662/jmaj.2024-0182

**Published:** 2025-03-28

**Authors:** Yu Michimuko-Nagahara, Yu Nakagama, Marvin Stanley Rodriguez, Natsuko Kaku, Yuko Nitahara, Katherine Candray, Evariste Tshibangu-Kabamba, Shinjiro Hamano, Kenji Hirayama, Akira Kaneko, Junko Nakajima-Shimada, Yoko Onizuka, José Eduardo Romero, José Ricardo Palacios, Carmen Elena Arias, William Mejía, Ricardo Cardona Alvarenga, Yasutoshi Kido

**Affiliations:** 1 Department of Parasitology & Research Center for Infectious Disease Sciences, Graduate School of Medicine, Osaka Metropolitan University, Osaka, Japan; 2 Department of Parasitology, Graduate School of Biomedical Sciences, Nagasaki University, Nagasaki, Japan; 3 Centro de Investigación y Desarrollo en Salud (CENSALUD), Universidad de El Salvador, San Salvador, El Salvador; 4 Department of Immunogenetics, Institute of Tropical Medicine (NEKKEN), Nagasaki University, Nagasaki, Japan; 5 School of Tropical Medicine and Global Health, Nagasaki University, Nagasaki, Japan; 6 Department of Molecular and Cellular Parasitology, Graduate School of Health Science, Gunma University, Gunma, Japan; 7 Ministerio de Salud, San Salvador, El Salvador; 8 Centro Nacional de Investigaciones Científicas de El Salvador, San Salvador, El Salvador; 9 Ministerio de Educación, Ciencia y Tecnología, San Salvador, El Salvador

**Keywords:** vectorial zoonosis, *Trypanosoma cruzi*, *Triatoma dimidiata*, blood feeding source, reservoir, One Health approach

## Abstract

**Introduction:**

Chagas disease is one of the most critical of the neglected tropical diseases in Latin America where it poses a serious public health issue. However, the current burden of vectorial transmission from natural reservoirs to humans is unclear. This study aimed to clarify the active mode of transmission to humans disentangled from the feeding pattern of *Triatoma dimidiata* (*T. dimidiata*) infected by *Trypanosoma cruzi* (*T. cruzi*).

**Methods:**

A total of 1,376 *T. dimidiata* specimens were collected across the 14 departments of El Salvador. From these specimens, 135 midgut samples from 37 households in eight departments were positive for *T. cruzi* (n = 135/1,376; 9.8% [95% confidential interval (CI): 8.35%-11.5%]). Using a universal vertebrate primer, vertebrate blood sources were positively identified by next-generation sequence analysis of deoxyribonucleic acid (DNA) extracted from the midgut contents of *T. dimidiata.*

**Results:**

A total of 13 vertebrates were detected as blood sources; humans, and five domestic, three synanthropic, and four sylvatic species. Triatomines identified as having fed on human blood accounted for approximately 67% (n = 90/135 [95% CI: 58.3%-74.1%]) of the samples analyzed.

**Conclusions:**

In this study, a holistic understanding of the feeding patterns of *T. cruzi*-positive *T. dimidiata* in El Salvador is dated. The detection of human DNA in the midgut contents of* T. dimidiata* indicated the possibility of active vectorial transmission to humans.

## Introduction

Chagas disease is a vectorial zoonosis caused by the protozoan *Trypanosoma cruzi* (*T. cruzi*) and is the most critical of the neglected tropical diseases in Latin America ^(1)^. A triatomine bug, *Triatoma dimidiata* (*T. dimidiata*), is a blood feeding vector of the parasite *T. cruzi*, is known to feed on the blood of >200 vertebrate species, including mammals, birds, amphibians, and reptiles ^(2), (3), (4)^. These vertebrates are highly variable in terms of their competency in acquiring *T. cruzi* infection, ranging from highly competent mammals to avian species with low competency. Highly competent known hosts play the role of ‘reservoir hosts’ and are responsible for facilitating the transmission of *T. cruzi* ^(5)^. Indeed, several studies have reported that the risk of *T. cruzi* infection in humans parallels the prevalence of trypanosome infection in reservoir hosts, principally *T. cruzi*-infected domestic animals such as dogs ^(6), (7), (8)^. Consequently, holistic monitoring of the wide variety of reservoir hosts would facilitate the development of an effective Chagas disease control strategy.

Among the Latin American countries where Chagas disease is endemic, El Salvador has the highest levels of endemicity, with circulating *T. cruzi* is mainly classified as type 1 in the Discrete typing units (DTU) classification ^(9)^. Since the 1970s, the government of El Salvador and the Initiative for Chagas Disease Control in Central America developed a vector control program that resulted in a dramatic reduction in human *T. cruzi* seroprevalence; from 20·5% in 1970 to 3·0% in 2000 ^(10), (11)^. A decline in the number of new-onset Chagas disease diagnoses has also been observed ^(12)^. However, a recent epidemiological survey targeting triatomine vectors from 2018 to 2020 reported that the prevalence of *T. cruzi* among vectors collected from domestic areas was as high as 10·0%. Taken together, the findings suggest that active vectorial transmission may unfortunately remain a clear and present danger in the region.

Despite the potential resurgence of the disease, a holistic picture of the transmission network unique to the region, i.e., natural reservoir that sustain transmission, is still unclear and in need of attention. Identifying the blood meal-source is considered to be an optimal approach for uncovering the parasite transmission network ^(13)^. Recently, quantitative assessment of the triatomines’ feeding behaviors using next-generation sequencing (NGS) platforms has enabled researchers to more comprehensively assess the differing intensities of contacts between the vector and various hosts ^(14)^. For example, using this approach to target Chagas disease showed that human encroachment into natural areas, such as engagement in outdoor activities, might increase the incidence of blood feeding by triatomine vectors, increasing the unexpected risk of disease transmission to humans ^(15)^.

The “One Health” approach emphasizes that the health of humans, animals, and ecosystems are interdependent, and that effective disease prevention, control, and management require cooperation across these domains. The present study aimed to investigate the heterogeneity of potential reservoir hosts of *T. cruzi* in El Salvador, employing an NGS-based *T. cruzi*-positive* T. dimidiata* blood meal-source analysis. This approach provides valuable insights for developing more effective and comprehensive strategies for Chagas disease control in the region.

## Materials and Methods

### Sample collection and specimen preparation

The *T. dimidiata* specimens used in this study partially overlap with a subset of vectors reported in our previous study ^(16)^. From December 2018 to September 2019, a total of 1,376 *T. dimidiata* were collected from 105 households across all 14 departments of El Salvador, specifically from the domestic and peri-domestic areas surrounding these households. Out of the collected 1,376 triatomines, a total of 135 triatomines which were confirmed to be positive for* T. cruzi,* were analyzed for this study. These triatomines were collected from 37 households across eight departments, and their positivity for* T. cruzi* was validated through microscopic examination and polymerase chain reaction (PCR) testing ^(16)^. DNA was extracted from the dissected midgut contents using a Qiagen DNAeasy Blood & Tissue kit (Qiagen, Venlo, The Netherlands). To address contamination concerns related to human DNA and prevent cross-contamination between samples, the operator wore gloves and safety goggles while handling the midgut contents and ensured that dissection tools were replaced for each sample. In morphological species determination, we distinguished particularly similar species by examining characteristics such as the color of the corium, ventral coloration, the setae around the abdomen, and other features ^(17), (18)^. For collected triatomines, the household from which they were collected was recorded and registered. The demographic parameters of the triatomines were described based on morphological analysis and the collected records. To ensure a more accurate characterization of the investigated households, we conducted a questionnaire on housing construction materials as well as the presence of any animals in the households at the time of collection.

### Molecular analysis for blood source detection

A two-step tailed PCR was performed to identify the blood source and assess the diversity among vector species. The primer set for the first step (F-5'-CAAACTGGGATTAGATACC-3', R-5'-AGAACAGGCTCCTCTAG-3') was used to amplify a 145 base pair (bp) mitochondrial 12S ribosomal ribonucleic acid (rRNA) gene region that targets a vertebrate-specific orthologous fragment ^(19)^. The first PCR amplification was performed in a 50 μl reaction volume containing 1 μl of DNA, the reaction mixture of the KAPA HiFi HotStart Kit (Kapa Biosystems, Wilmington, MA), PCR-grade water, and primer (5 μM). Thermocycling conditions consisted of denaturation at 95°C for 3 minutes, followed by 35 cycles of 95°C for 30 seconds, 63°C for 30 seconds, and 72°C for 30 seconds, and a final extension step of 72°C for 5 minutes. The second primer set converts the amplicon into a library ready for performing MiSeq sequencing using the Nextera XT Index Kit (Illumina, San Diego, CA); performing this step permits multiplexing of individual triatomines for the NGS analysis. The second PCR amplification was performed in a 50 μl reaction volume containing 5 μl of purified deoxyribonucleic acid (DNA) product from the first PCR (DNA), the reaction mixture of the KAPA HiFi HotStart Kit, PCR-grade water, and Nextera XT Index Kit (N7xx, S5xx). Thermocycling conditions for this step consisted of denaturation at 95°C for 3 minutes, followed by eight cycles of 95°C for 30 seconds, 55°C for 30 seconds, and 72°C for 30 seconds, and a final extension step of 72°C for 5 minutes. Amplicon sequencing was performed on an Illumina MiSeq using a PE300 kit (Illumina, San Diego, CA).

All sequencing data were analyzed using CLC Genomics Workbench version 21.0.4 (https://digitalinsights.qiagen.com/). Data was handled as follows. First, we performed normalization and trimming to extract randomly around 50,000 reads and to trim sequences based on the adapter region. Then, raw sequences were clustered against a custom 12S reference database. The database contains 136 vertebrate sequences, including 124 sequences from the vertebrate database used in a previous study conducted in Panama ^(19)^ and 12 newly added vertebrate sequences downloaded from GenBank (Supplemental File S1). We used the operational taxonomic unit (OTU) clustering method to cluster the obtained sequences. Simultaneous filtering was performed to remove low-quality reads and chimeric sequences (if present). Sequences that matched with >97% identity were used for vertebrate identification. The non-aligned reads, which accounted for 29% of the total reads (888,592/3,048,772), were attributed to PCR error since they did not match any of the sequences registered in GenBank. We manually curated these non-aligned sequences and discarded them from further analysis. A schematic diagram of the bioinformatics pipeline is provided in Supplemental Figure 1.

### Data analysis

We described the relative abundance of the obtained reads among the various vertebrate species based on the proportion of the mapped reads. In order to normalize the obtained sequences, the mapped reads derived from an individual were converted into proportions for each species. The sum of these proportions was then used to determine the relative abundance of blood- sources.

The 95% confidence interval (95% CI) of proportions in this study was estimated using the Agresti and Coull method, a widely recommended approach due to its balance between precision and interval coverage. This method is less conservative than the Clopper-Pearson (exact) interval and offers better performance in terms of interval accuracy compared to the Wald interval ^(20)^. Therefore the DescTools package of R was used ^(21)^.

To evaluate differences in blood meal source composition by life stage and capture location of *T. dimidiata,* the number of gene fragments was organized into each group. The Wilcoxon rank-sum test was used for statistical analysis. Then, to determine whether the animals were or were not the blood source of each triatomine, we used a relative abundance value of 2% as a cutoff value for the total reads for an individual triatomine.

Descriptive analysis of the variables recorded for the parasite infection rate for each household and each blood feeding source of triatomines was conducted. Specifically, we divided the *T. dimidiata* specimens into two groups based on the *T. cruzi* infection rate. One group comprised *T. dimidiata* specimens that were collected from households with a *T. cruzi* infection rate <33% (n = 79), and the other group comprised *T. dimidiata* specimens collected from households with a *T. cruzi* infection rate 33% and over (n = 52). The rationale for using 33% as the threshold for the grouping was that the median *T. cruzi* infection rate of 37 households was 33%. To study the relationship among the variables of interest, a Mann-Whitney test was used. In addition, Spearman’s rank correlation coefficient analysis was also used to test the correlation between parasite infection rate by household and the relative abundance of less likely hosts. Statistical analysis was performed using GraphPad Prism for Windows 64-bit, version 9.0 (GraphPad Software, La Jolla CA). A p-value of less than 0.05 was considered statistically significant.

## Results

### Demographic characteristics of targeted ***T. dimidiata***

We analyzed 135 *T. dimidiata* specimens, collected from 37 households, that were positive for *T. cruzi*. Their life stage, sex, and collection site are shown in Table 1. The specimens were collected in a total of eight different departments, with the greatest numbers collected from San Miguel (33%), Usulután (24%), Chalatenango (16%), and Ahuachapán (8·9%), which together accounted for 82% of the total. Further, 81% (30/37) of participating households responded to the questionnaire survey, the results of which are summarized in Table 2. Importantly, all of the households surveyed, from which *T. dimidiata* were collected, had living spaces that were shared with at least one animal. Chickens were the most common, sharing the living space with their owners in 26/30 (87%) of the households, followed by dogs (23/30, 77%). In terms of housing material, 53% (16/30) of households had mudded walls, 43% (13/30) had tinned roofs, and 63% (19/30) had soil floors. The majority of houses were made, at least in part, of housing material that was conducive to *T. dimidiata* infestation ^(22)^.

**Table 1.** Demographic Characteristics of Analyzed *T. dimidiata*.

Demographic characteristics of analyzed *T. dimidiata*	Frequency	(%)	95% confidence interval
**Life stage, n = 135**			
Nymph	76	(56)	(48-64)
Adult	59	(44)	(36-52)
Sex (adult stage) *, n = 59			
Male	29	(49)	(37-62)
Female	30	(51)	(38-63)
**Collection site, n = 135**			
Domestic	108	(80)	(72-86)
Peri-domestic	27	(20)	(14-28)
**Department, n = 135**			
Ahuachapán	12	(8·9)	(5·0-15)
Cabañas	9	(6·7)	(3·4-12)
Chalatenango	22	(16)	(11-24)
Cuscatlán	3	(2·2)	(0·5-6·6)
Morazán	3	(2·2)	(0·5-6·6)
San Miguel	45	(33)	(26-42)
San Salvador	9	(6·7)	(3·4-12)
Usulután	32	(24)	(17-31)

*Only adults were sexed (n = 59).

**Table 2.** Characteristics of Targeted (Investigated) Households.

Characteristics	Frequency	(%)	95% confidence interval
Presence of animal*, n = 30			
Dog	23	(76)	(59-89)
Chicken	26	(87)	(70-95)
Pig	2	(6·7)	(0·8-22)
**Presence of chicken coop, n = 26**			
No	17	(65)	(46-81)
Yes	9	(35)	(19-54)
**Wall material, n = 30**			
Mud	16	(53)	(36-70)
Block	6	(20)	(9·1-38)
Bamboo	5	(16)	(6·8-34)
Wood	1	(3·3)	(0·0-18)
Brick	1	(3·3)	(0·0-18)
Not available	1	(3·3)	(0·0-18)
**Roof material, n = 30**			
Tin	13	(43)	(27-61)
Tile	15	(50)	(33-67)
Cement	1	(3·3)	(0·0-18)
Not available	1	(3·3)	(0·0-18)
**Floor material, n = 30**			
Soil	19	(63)	(46-78)
Brick	11	(37)	(22-55)

*Total percentage exceeds 100% due to multiple animals in the household.

### Blood feeding source of ***T. cruzi***-positive ***T. dimidiata***

In order to identify the species of the feeding source, we assigned all of the sequences (i.e., a 145-bp sequence of the mitochondrial 12S rRNA amplicon), to an OTU, which is considered a basic unit in numerical taxonomy. Of the total sequence reads obtained from the amplified PCR products from 135 *T. dimidiata* midgut contents, 71% (2,160,180/3,048,772) were successfully aligned using the vertebrate 12S rRNA sequence library, with alignments ranging from 36% to 99% per sample. Regarding the non-aligned reads that were attributed to PCR error, we manually curated sequences and discarded them from the analysis.

**Figure 1.**
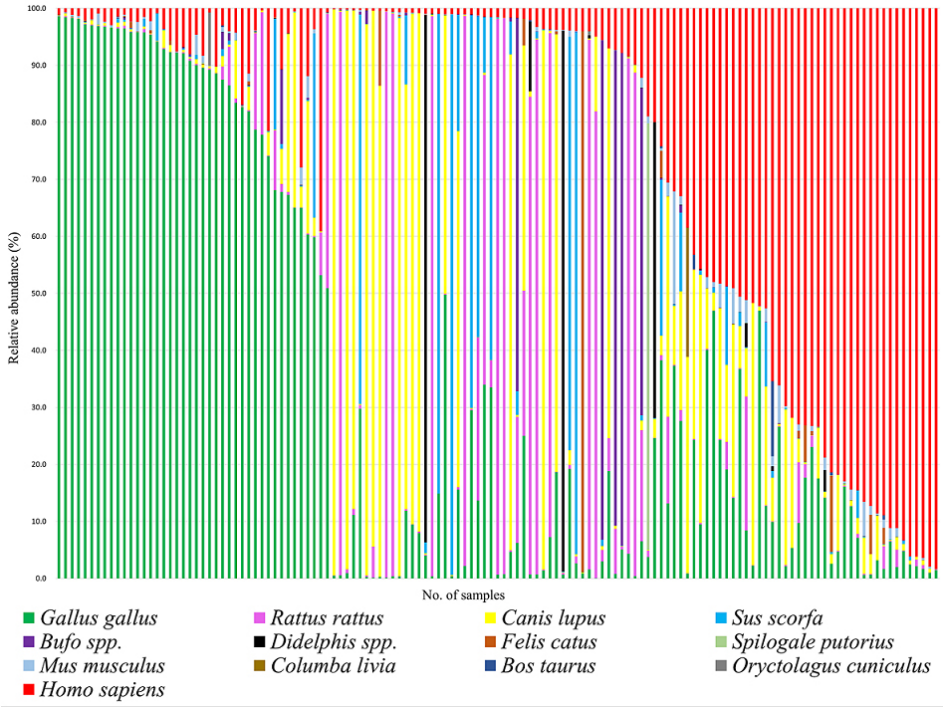
Relative abundance of target-vertebrate animal in each vector. Each bar graph represents a sequence derived from an individual *T. dimidiata*. The bar graph is color-coded by species and is expressed as a percentage of the number of reads of each triatomine. *T. dimidiata: Triatoma dimidiata*.

Thirteen vertebrate species were identified as being the vector’s blood meal sources (Figure 1). Among these, six species were newly identified as potential blood sources for *T. dimidiata* in the region (i.e., bufo, cat, skunk, pigeon, cow, and rabbit), while seven species had been previously reported, including chicken, human, dog, black rat, pig, mouse, and opossum ^(23), (24), (25)^. In decreasing order of relative abundance, chickens, humans, dogs, and black rats accounted for the majority of the blood meals, comprising over 80% of the total mapped reads. Although the predominant blood sources, such as chickens, humans, and dogs, were consistent with previous studies ^(24)^, our findings reveal that some livestock and sylvatic animals also serve as significant feeding sources for the vector. As shown in Figure 1, multiple blood meal sources were detected within individual triatomines, with an average of 2·4 ± 1·1 (1-6) blood sources per triatomine, highlighting the complex feeding behavior of *T. dimidiata*.

**Figure 2.**
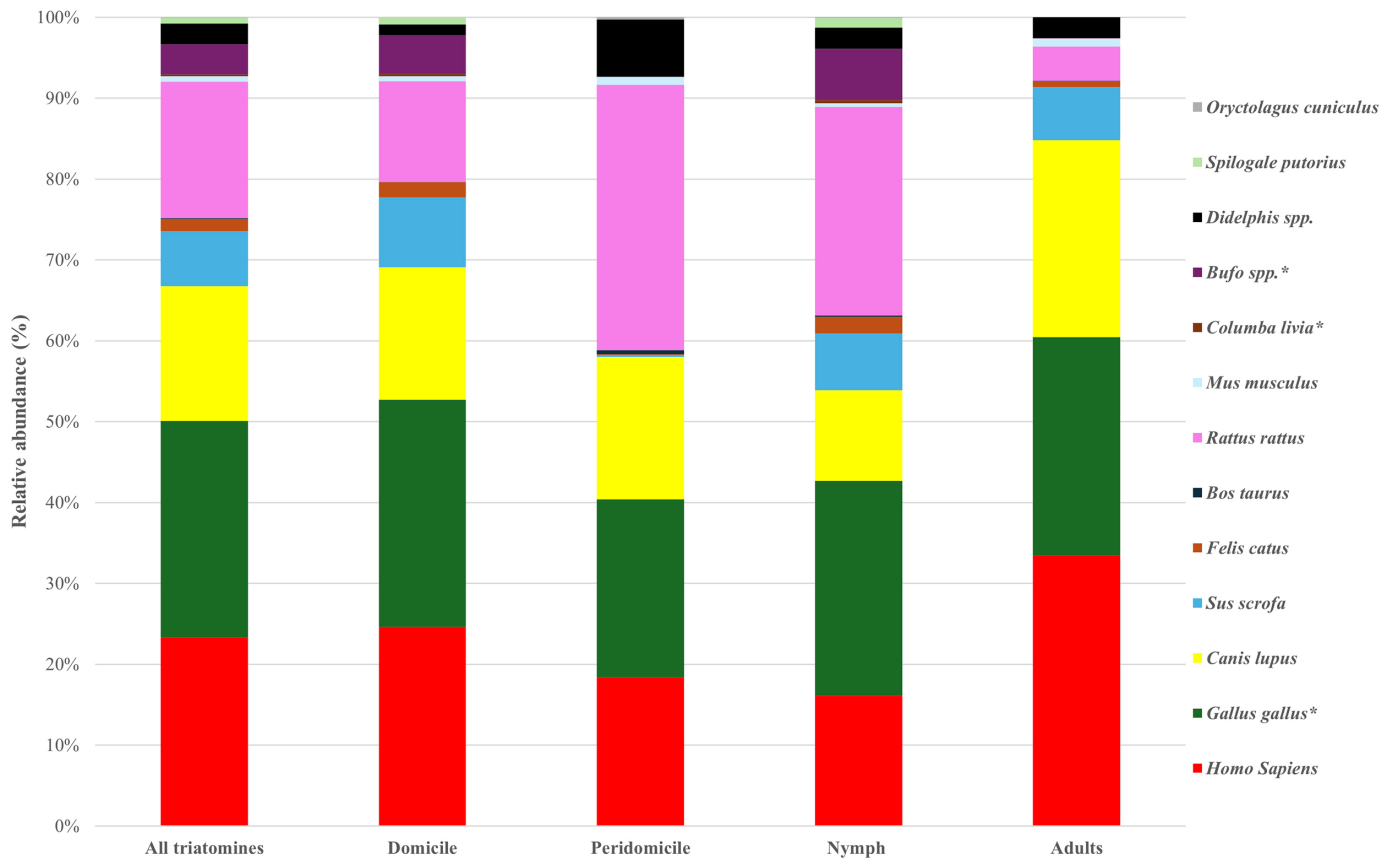
Proportions of blood source species in all triatomines and four categorized groups based on capture location and life stage. The proportions of blood source species were calculated based on the absolute number of sequences. A significant difference was observed in the components of blood source species between domicile and peridomestic groups (Wilcoxon rank-sum test, p = 0.006). * Less likely host

Figure 2 illustrates the proportions of blood sources for all 135 triatomines, as well as for those grouped by capture location and life stage. Statistical analysis using the Wilcoxon test revealed significant differences in blood source proportions between domicile-derived and peridomicile-derived triatomines (p = 0.006), while no significant differences were observed with respect to life stage (p = 0.147). The Shannon diversity index values were H’ = 1.79 for all triatomines, H’ = 1.82 for the nymph group, H’ = 1.53 for the adult group, H’ = 1.76 for the domicile-derived group, and H’ = 1.63 for the peridomestic-derived group. Notably, the overall Shannon-Wiener diversity index of 1.79 for all triatomines is consistent with previous studies ^(13)^, further illustrating the diverse feeding behavior of *T. dimidiata*.

**Figure 3.**
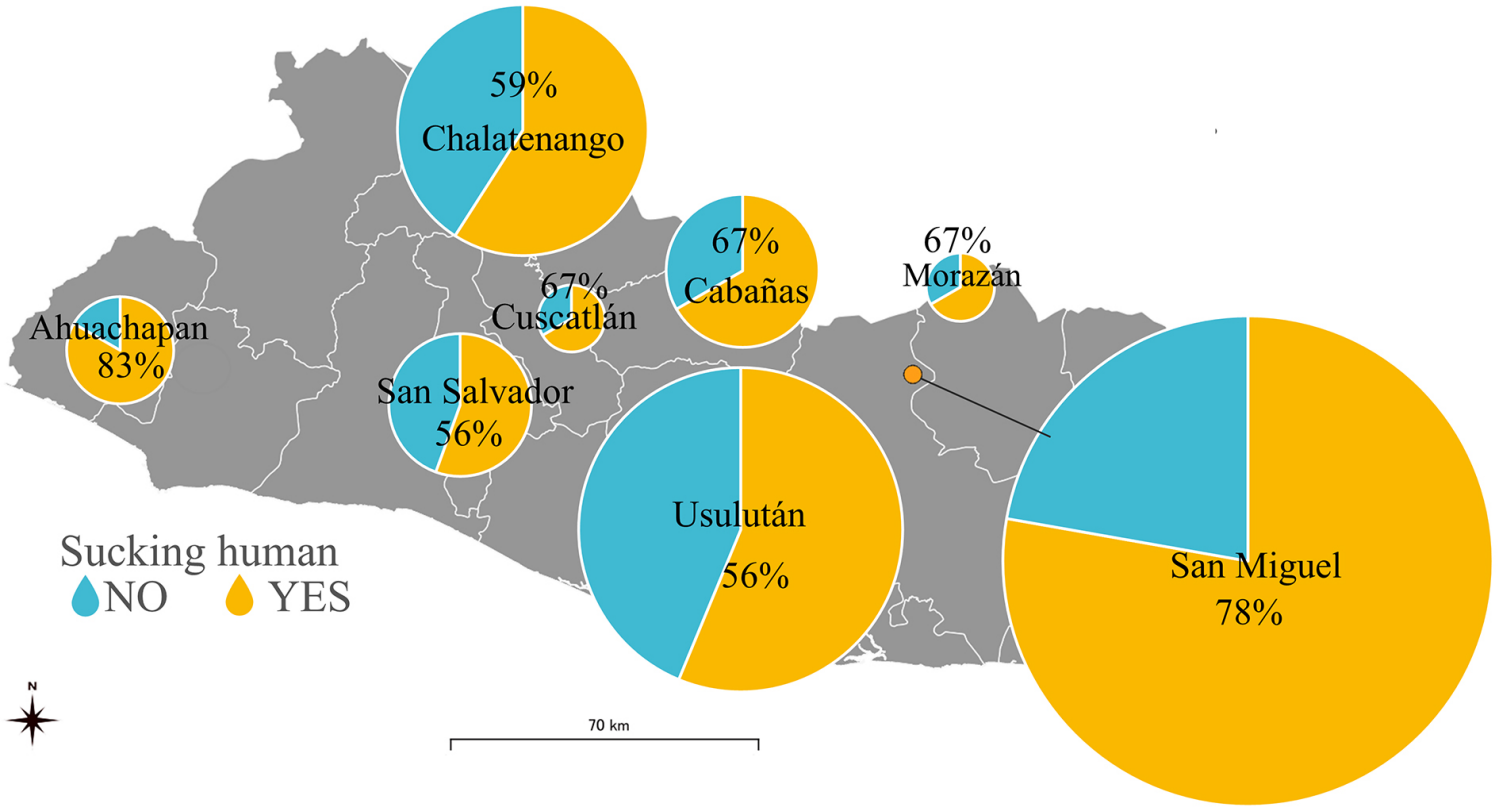
Human blood ingestion rate for different geographical locations. The radius of the pie chart for each department reflects the number of triatomines.

To investigate the feeding preference of the vectors, we calculated the detection rate of the animal in the total population of the vectors, using a 2% cutoff value for the presence or absence of the feeding source (n = 135) (Table 3). Importantly, human gene fragments were detected in 90 triatomines, which is equivalent to 67% of the total population. The geographical distribution of the detection rate of human blood at the department level is shown in Figure 3. The detection of human blood from *T. dimidiata* was confirmed in all eight departments, although the rate varied from 33% to 83% (average = 61%) per department.

**Table 3.** Detection Rate per Species.

Species	Frequency (n = 135)	(%)	95% confidence interval
*Gallus gallus* (Chicken)*	105	(78)	(70·0-84·0)
*Homo sapiens* (Human)	90	(67)	(58·3-74·1)
*Canis lupus* (Dog)	57	(42)	(34-51)
*Rattus rattus* (Black rat)	37	(27)	(20-36)
*Sus scrofa* (Pig)	22	(16)	(11-24)
*Mus musculus* (Mouse)	13	(9·6)	(5·6-16)
*Bufo* spp*.* (Bufo)*	8	(5·9)	(2·9-11)
*Felis catus* (Cat)	8	(5·9)	(2·9-11·4)
*Didelphis* spp*.* (Opossum)	6	(4·4)	(1·9-9·6)
*Bos taurus* (Cow)	2	(1·5)	(0·07-5·6)
*Spilogale putorius* (Skunk)	1	(0·7)	(0·00-4·5)
*Columba livia* (Pigeon)*	1	(0·7)	(0·00-4·5)
*Oryctolagus cuniculus* (Rabbit)	1	(0·7)	(0·00-4·5)

*Less likely host

**Figure 4.**
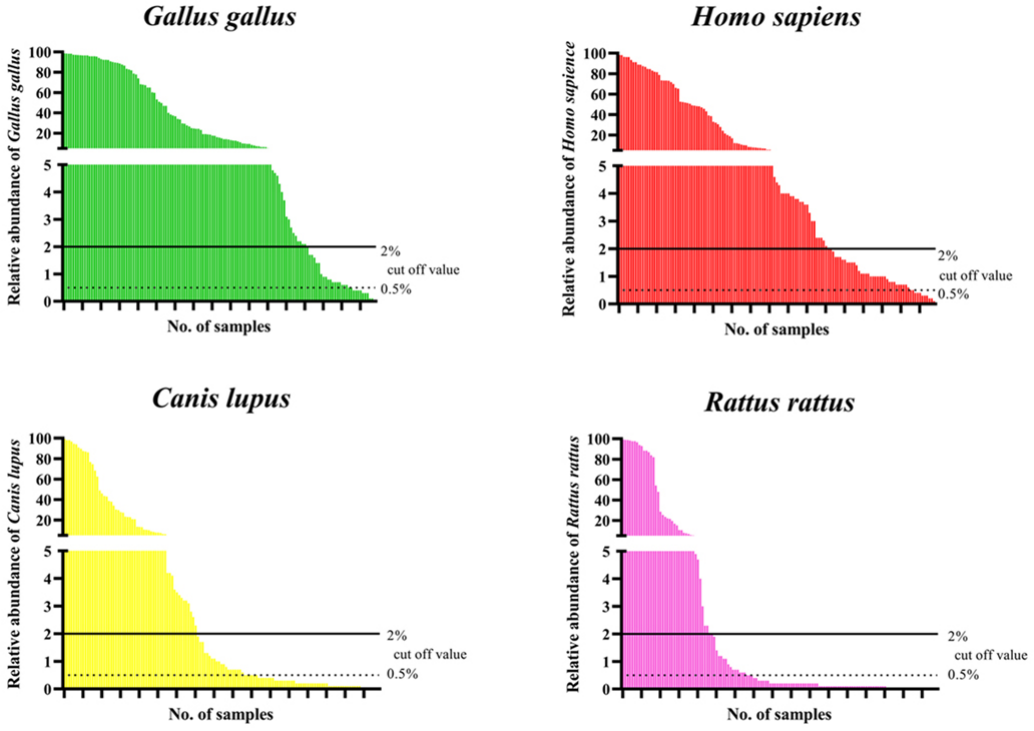
Relative abundance of four representative vertebrates in each sample. The number of sequences derived from individual vectors is used as the denominator, and each bar graph shows the sequence percentages for each animal in decreasing order.

The relative abundance values of each vertebrate varied per triatomine. Human DNA was detected from all 135 individual triatomines ranging from 0.1% to 98%, but 44 (33%) showed less than 2%, which was the cutoff value adopted in this study. At a cutoff value of 0.5% adopted in other studies, 35 (26%) individuals showed a relative abundance between 2% and 0.5%. Such a phenomenon was also observed in non-human vertebrates; for example, 19 (14%) in chickens, 26 (19%) in dogs, and 17 (13%) in black rats, with each vertebrate having genome fragments between 2% and 0.5% (Figure 4).

### Role of less likely vertebrate hosts serving as blood meals

**Figure 5.**
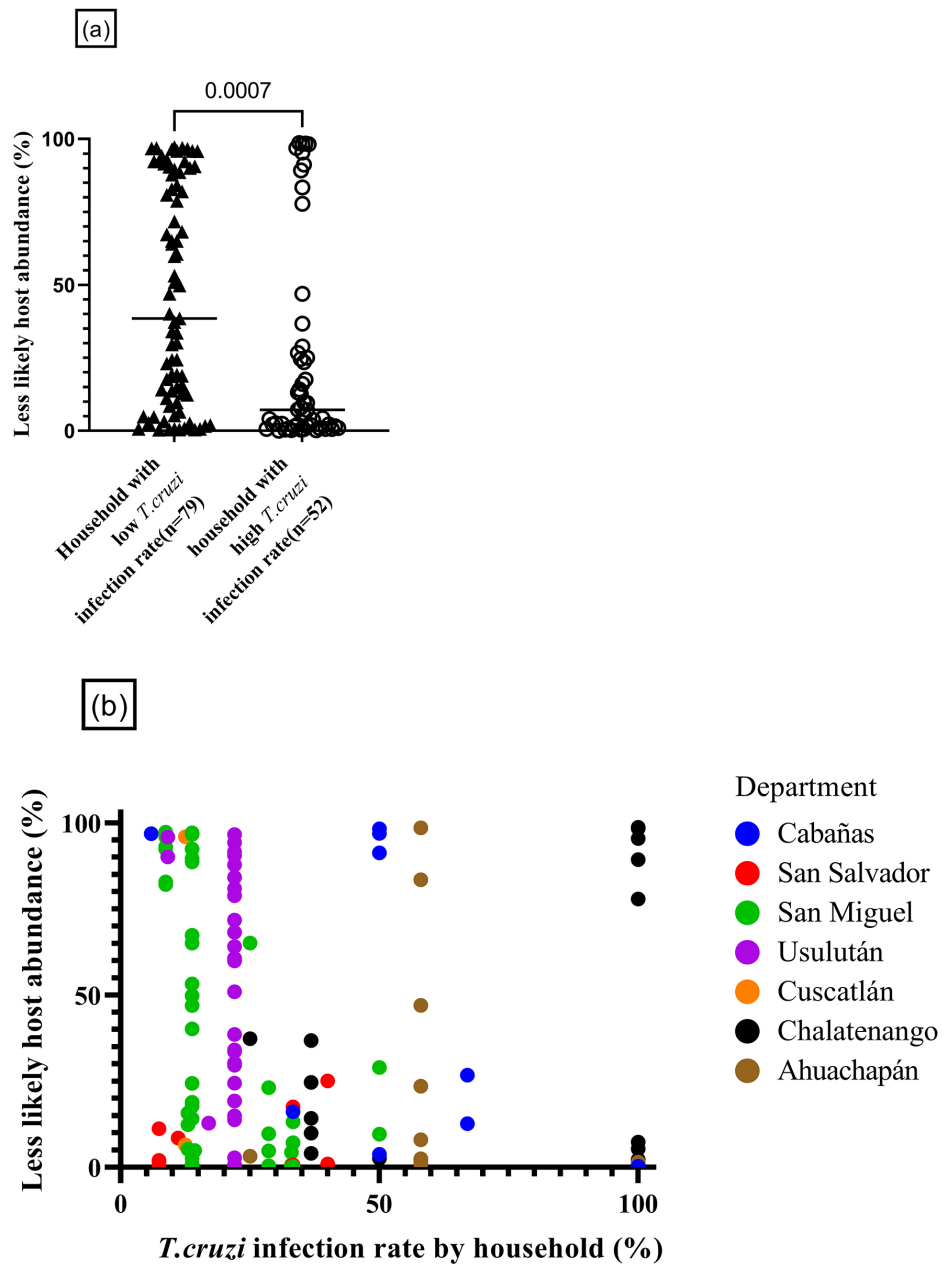
Implications of less likely hosts on *T. cruzi* infection rate. (a) Statistical considerations in the relative abundance of the less likely host (n = 131) We compared the relative abundance of less likely hosts with three vertebrates in two groups. The black triangle group has a *T. cruzi* infection rate per household of less than 33% (n = 79); the other has a *T. cruzi* infection rate per household of 33% and over (n = 52). One plot represents an individual vector. A significant difference was observed in less likely host abundance between the two groups (Mann-Whitney test, p = 0.0007). (b) Household variety of *T. cruzi* infection rate and relative abundance of less likely hosts (n = 131). We determined the correlation matrix for *T. cruzi* infection rate and less likely host abundance (n = 131). One plot represents an individual vector. A weak negative correlation was observed (Spearman’s correlation coefficient, *r*_s_ = −0.28, p = 0.001) between the infection rate of collected vectors calculated in each household and less likely host abundance. *T. cruzi: Trypanosoma cruzi*.

Since avian, amphibian, and reptile species are known to be low competency for *T. cruzi*, they are consequently less likely to contribute to the pathogen transmission. Several mathematical models have shown that the presence of such hosts may reduce transmission efficiency, a concept known as the "dilution effect" hypothesis, which has been explored in other insect-borne diseases ^(26), (27)^. However, no empirical data have yet confirmed this hypothesis for *T. cruzi* transmission. Thus, we sought to investigate whether the feeding behavior of *T. dimidiata* on these less competent hosts has any impact on the vector’s transmission rate of *T. cruzi*. As a results, households with lower vector *T. cruzi* infection rates (less than 33%) showed higher abundance in less likely hosts-derived reads among the feeding source of infested vectors, compared to households with high vector *T. cruzi* infection rates (33% or over) (79 vs 52 triatomines, p = 0.0007) (Figure 5a). Furthermore, a weak but significant, negative correlation was found in the *T. cruzi* infection rate at the household level and the relative abundance of less likely hosts (Spearman’s correlation coefficient, *r*_s_ = −0.28, p = 0.001) (Figure 5b). In total, feeding behavior toward less likely hosts (i.e., sharing living space with chickens) was associated with suppressed within-household vector-borne transmission.

### Feeding and possible pathogen transmission network

**Figure 6.**
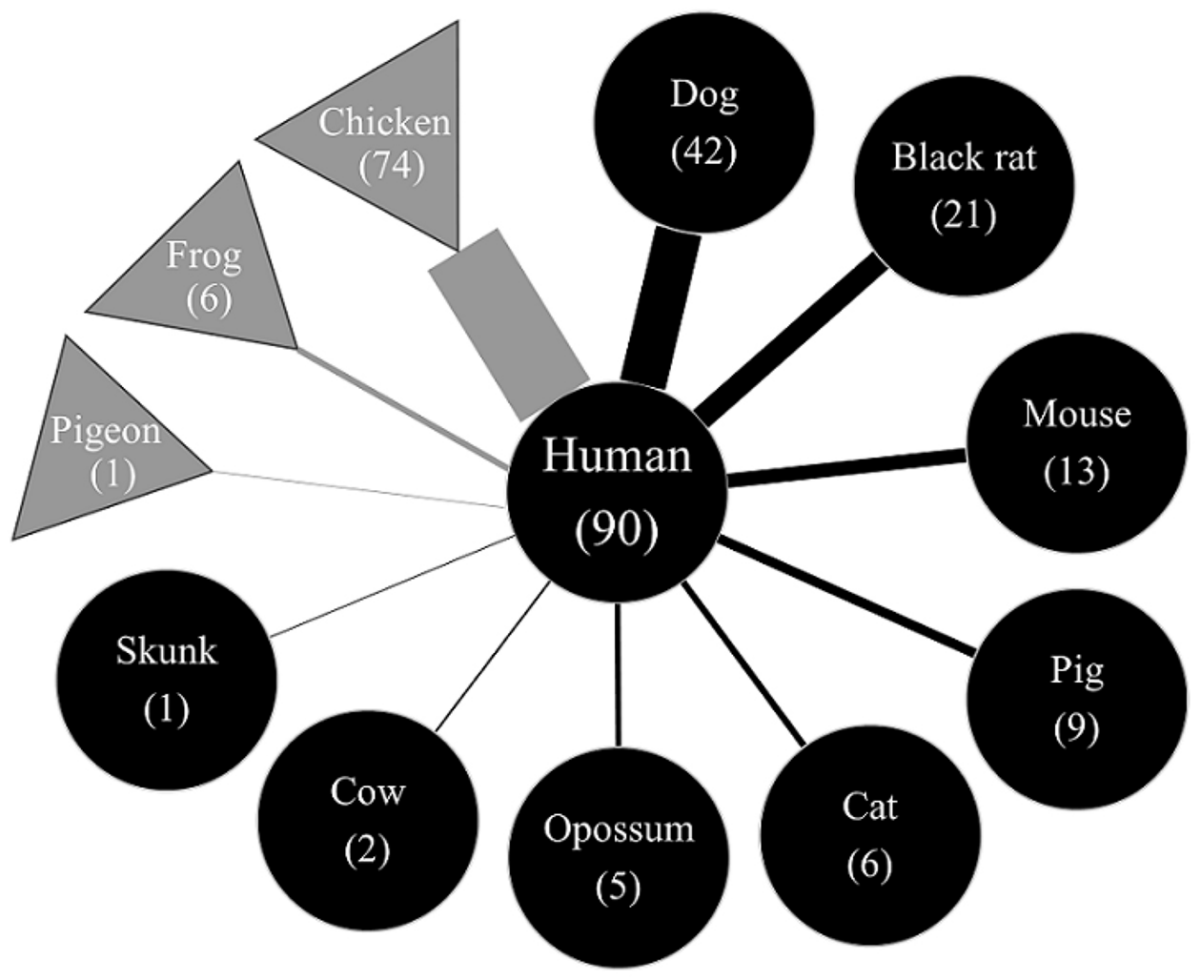
Feeding and possible parasite transmission network. Feeding networks were described to visualize possible transmission pathways of *T. cruzi* and the frequency of the respective blood-feeding sources. Animals indicated by black circles are known hosts and animals indicated by gray triangles are less likely hosts. The thickness of the line connecting the vertebrates reflected the detection frequency of the vertebrates. *T. cruzi: Trypanosoma cruzi*.

To identify the vertebrate(s) involved in the transmission of pathogens to humans, we elucidated the possible pathogen network of vectorial transmission by identifying the co-detected animals of *T. dimidiata* that were considered positive for human blood (n = 90) (Figure 6). In decreasing order, the upper quartile of the blood source detection frequency consisted of co-detection with chickens, dogs, black rats, pigs, opossums, and bufo frogs. Dogs are the most detected blood source of *T. dimidiata* that were positive for human blood, suggesting that parasite transmission could occur between humans and dogs more frequently than among other known host animals that share dwellings with humans.

## Discussion

In this study, we conducted a comprehensive rRNA gene-based blood meal source analysis of *T. cruzi*-positive *T. dimidiata* that were collected from different regions throughout El Salvador to clarify the full range of source vertebrates. Since the reservoir species have a significant effect on Chagas disease dynamics ^(28)^, our findings are considered to be important for building comprehensive future control programs.

The most frequently encountered blood feeding source among known hosts was humans. The frequent (67%) detection of human-derived rRNA gene fragments in the midgut contents of *T. cruzi*-positive *T. dimidiata* suggested significant human-parasite contact at the vector level. Cataloging the potential reservoir species may allow identification of their active involvement in the transmission of Chagas disease and clarify their contribution to the recent resurgence of the disease in the country. This observation emphasizes the need to re-intensify vector-targeted prevention and control measures in the region.

As shown by our data, the detection of chicken gene fragments was considerably higher in El Salvador than in other Central American countries ^(23), (24), (25)^. Since birds and amphibians are known to have low competency in transmitting *T. cruzi,* the proportion of the blood meal derived from less likely hosts (i.e., chickens, bufos, and pigeons) were related to suppression in the *T. cruzi* infection rate among regional *T. dimidiata* (Figure 5b). Thus, the farming of chickens may have a combined effect on the mitigation of Chagas disease risk, not only by socio-economically improving a person’s status through increased income ^(29)^ but also by increasing the proportion of blood feeding on chickens.

The high rate of co-detection of human and dog rRNA gene fragments suggested that parasite transmission might occur frequently between the two vertebrates, as has been reported in other countries ^(14), (30), (31), (32)^. In addition, the elevated detection of gene fragments from dogs and humans appears to be closely linked to the habitats of triatomines, as shown in the demographic data of the analyzed population (Table 2). Since dogs are both more sensitive to parasites than humans and have shorter life spans, they are considered to be important targets for both monitoring and intervention in order to reduce human transmission ^(6), (31), (33)^. Zoonotic helminths, such as ascarids, hookworms, and roundworms, are also shared between humans and dogs, and therefore, anthelmintic drugs have been administered to dogs for the purpose of preventing human transmission ^(34)^. Previous studies have shown that the anthelmintic agents that disrupt the nervous systems of insects, such as deltamethrin and fluralaner, demonstrated long-term (up to 6 months) insecticidal effects on triatomines that have fed on the blood of dogs medicated with these agents ^(6)^. Although these drugs cannot fully protect dogs from *T. cruzi* infection ^(35)^, they may indirectly impact the dog-human transmission axis through their insecticidal effect against triatomine vectors.

In conclusion, our data revealed an ever-wider variety of potential reservoirs, as well as less likely hosts. In addition, the blood source analyses performed in this study are considered to be highly relevant to vectorial *T. cruzi* transmission through *T. dimidiata* in El Salvador. Understanding the diversity of the ecosystem surrounding *T. dimidiata* will help to formulate effective control strategies, based on the concept of “One Health” in El Salvador.

### Limitations

While this study provides key insights into the feeding behavior of* T. dimidiata*, several limitations must be acknowledged. First, the sampling method focused on households with specific criteria ^(16)^: (1) natural material walls and (2) prior reports from the Ministry of Health confirming the presence of* T. dimidiata*. This introduces potential selection bias and limits the generalizability of our findings to broader regions with different environmental characteristics. Additionally, our exclusive analysis of parasite-positive triatomines restricts a holistic understanding of feeding behaviors in the general triatomine population. Finally, the cross-sectional design further limits the study’s ability to assess temporal feeding patterns, preventing insights into changes in feeding behaviors over time.

Our amplicon analysis determined the presence or absence of a blood source based on an empirical relative abundance cutoff value of 2%. Various cutoff values have been adopted in similar studies ranging from 0·5% to 10% ^(13), (14), (36)^. Using a rather low 2% cutoff value, we may have potentially overestimated the variety of blood sources in *T. dimidiata*. Difficulties in the cross-study comparisons of rates of detection of human DNA, due to aspects such as the arbitrary nature of cutoff settings, raise questions about the strength of associations between these rates and the local endemicity of Chagas disease. To better evaluate the strength of associations between human detection rates and the endemicity of Chagas disease, a longitudinal survey consisting of blood feeding behavior monitoring and novel case detection is warranted.

## Article Information

### Conflicts of Interest

None

### Sources of Funding

This research was supported by Science and Technology Research Partnership for Sustainable Development, Japan Agency for Medical Research and Development (under Grant Numbers JP21jm0110016 to Yasutoshi Kido and JP23wm0325049 to Yu Nakagama), Japan International Cooperation Agency and Japan Society for the Promotion of Science Kakenhi (22KK0279 to Yu Nakagama). The funding agencies had no role in conceptualizing this study.

### Acknowledgement

This work was a joint investigation conducted by El Salvadorian and Japanese research centers, part of Official Development Assistance from Japan, and was supported by Japan International Cooperation Agency (JICA). We are thankful to JICA El Salvador as well as Masako Shibata, Ui Yamada, Yoko Hamaguchi, and Hiroshi Kunikane for their support. We gratefully appreciate the field surveyors for their technical work. We would also like to thank Prof. Kiyoshi Kita for valuable discussions.

### Author Contributions

Yu Michimuko-Nagahara, Yu Nakagama, and Yasutoshi Kido designed the study. José Eduardo Romero, José Ricardo Palacios, Carmen Elena Arias, William Mejía, and Ricardo Cardona Alvarenga performed vector sampling. Yu Michimuko-Nagahara, Yu Nakagama, Katherine Candray, Yuko Nitahara, Evariste Tshibangu-Kabamba, and Marvin Stanley Rodriguez performed amplicon analysis. Yu Michimuko-Nagahara, Yu Nakagama, Natsuko Kaku, and Yasutoshi Kido wrote the manuscript and contributed to data analysis and interpretation. Shinjiro Hamano, Kenji Hirayama, Akira Kaneko, Yoko Onizuka, and Junko Nakajima-Shimada contributed to critical discussions of the manuscript.

### Approval by Institutional Review Board (IRB)

Not applicable

## Supplement

Supplemental Figure 1. A schematic diagram of the bioinformatics pipeline separating NGS-based amplicon analysis obtained from the abdominal specimens.
Raw data from 135 T. dimidiata were trimmed and filtered using CLC Genomic Workbench and then mapped to the 12S rRNA reference database. The remaining unmapped reads were queried by NCBI nt database (October 2021) to verify matches obtained from vertebrates.
NCBI: National Center for Biotechnology Information; NGS: next-generation sequencing; nt: nucleotide; rRNA: ribosomal ribonucleic acid; T. dimidiata: Triatoma dimidiata.

Supplemental File 1 Custom 12S reference database of 136 sequences based on sequences downloaded from GenBank.
