# Supplementary figures and images for: Natural Reservoir of Trypanosoma cruzi Found in Triatomines Targeting Humans: Results from Nation-wide Vector Surveillance in El Salvador
Source: JMA J. 2025 Mar 28;8(2):432–43. doi: 10.31662/jmaj.2024-0182 (PMC12095551; doi:10.31662/jmaj.2024-0182)

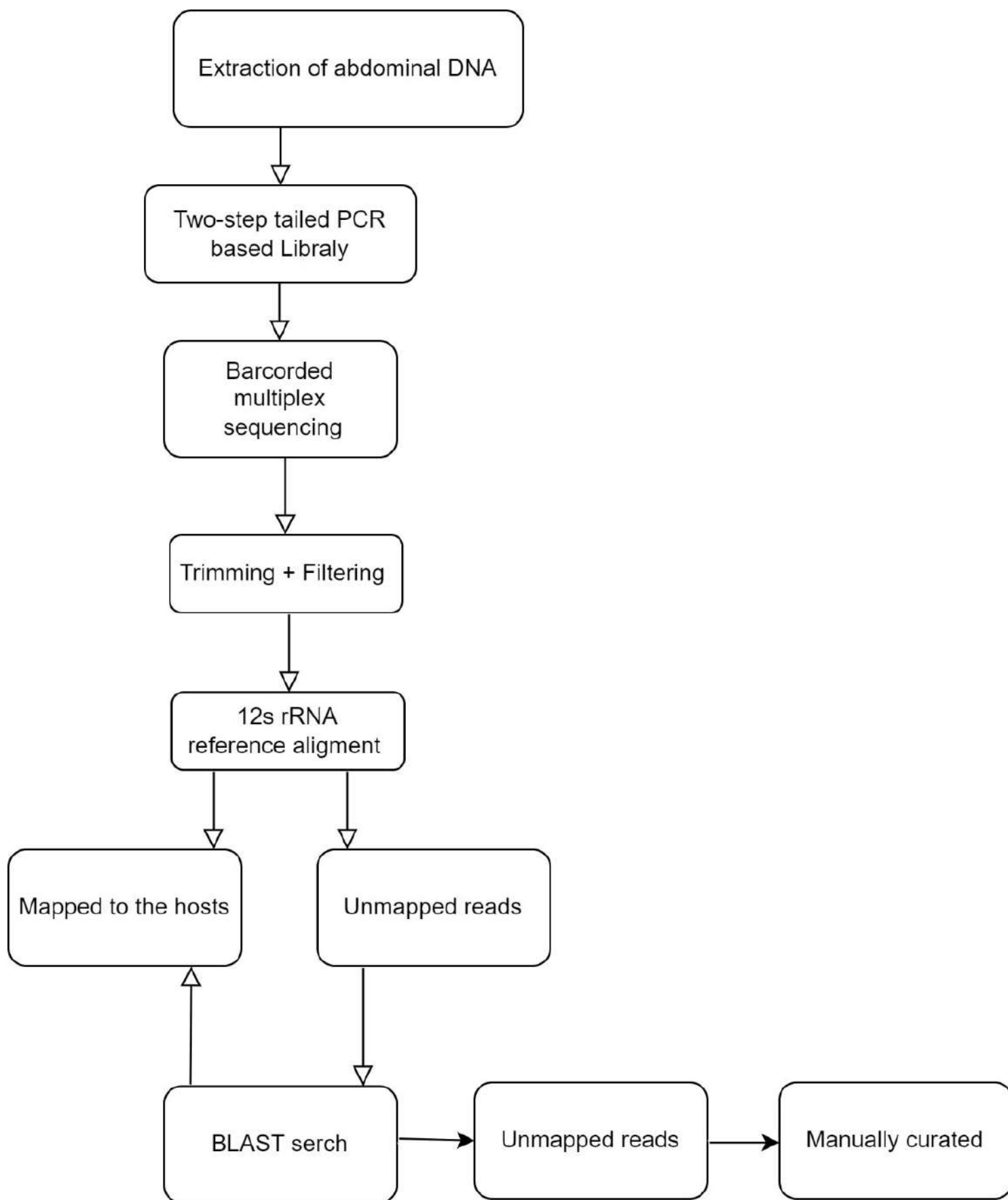

Supplement: Supplemental Figure 1. — A schematic diagram of the bioinformatics pipeline separating NGS-based amplicon analysis obtained from the abdominal specimens. Raw data from 135 T. dimidiata were trimmed and filtered using CLC Genomic Workbench and then mapped to the 12S rRNA reference database. The remaining unmapped reads were queried by NCBI nt database (October 2021) to verify matches obtained from vertebrates. NCBI: National Center for Biotechnology Information; NGS: next-generation sequencing; nt: nucleotide; rRNA: ribosomal ribonucleic acid; T. dimidiata: Triatoma dimidiata. [file 2433-3298-8-2-0432-s001.pdf]
