# Supplementary material for: Natural Reservoir of Trypanosoma cruzi Found in Triatomines Targeting Humans: Results from Nation-wide Vector Surveillance in El Salvador
Source: JMA J. 2025 Mar 28;8(2):432–43. doi: 10.31662/jmaj.2024-0182 (PMC12095551; doi:10.31662/jmaj.2024-0182)
Supplement: Supplemental File 1 — Custom 12S reference database of 136 sequences based on sequences downloaded from GenBank. [file 2433-3298-8-2-0432-s002.pdf]

1.>AY012095.1 Euphractus sexcinctus 12S ribosomal RNA gene, partial sequence; and tRNA-Val gene, complete sequence; mitochondrial

2.>AJ505826.1 Euphractus sexcinctus mitochondrial 12S rRNA gene, specimen voucher T-2351

3.>AF422863.1 Proechimys amphichoricus clone ALG 14040 12S ribosomal RNA gene, partial sequence; mitochondrial gene for mitochondrial product

4.>U12447.1 Proechimys longicaudatus mitochondrion 12S rRNA gene, partial sequence

5.>KF590707.1 Proechimys cuvieri 12S ribosomal RNA gene, partial sequence; mitochondrial

6.>AF422864.1 Proechimys simonsi clone JLP 11051 12S ribosomal RNA gene, partial sequence; mitochondrial gene for mitochondrial product

7.>EF405916.1 Bradypus variegatus isolate Bravar62PA 12S ribosomal RNA gene, partial sequence; mitochondrial

8.>Z48937.1 B.variegatus mitochondrial 12S rRNA gene

9.>AY012093.1 Choloepus hoffmanni 12S ribosomal RNA gene, partial sequence; and tRNA-Val gene, complete sequence; mitochondrial

10.>KR336793.1 Choloepus hoffmanni mitochondrion, complete genome

11.>EF405911.1 Cyclopes didactylus isolate Cycdid09PB 12S ribosomal RNA gene, partial sequence; mitochondrial

12.>EF405910.1 Cyclopes didactylus isolate Cycdid11PA 12S ribosomal RNA gene, partial sequence; mitochondrial

13.>NC_028574.1 Tamandua mexicana voucher MVZ_192699 mitochondrion, complete genome

14.>KT818551.1 Tamandua mexicana voucher MVZ_192699 mitochondrion, complete genome

15.>AF420045.1 Aotus azarai 12S ribosomal RNA gene, partial sequence; tRNA-Val gene, complete sequence; and 16S ribosomal RNA gene, partial sequence; mitochondrial

16.>AF069977.1 Aotus trivirgatus 12S ribosomal RNA gene, mitochondrial gene for mitochondrial RNA, complete sequence

17.>AF069972.1 Saguinus geoffroyi 12S ribosomal RNA gene, mitochondrial gene for mitochondrial RNA, complete sequence

18.>AF069973.1 Saguinus oedipus 12S ribosomal RNA gene, mitochondrial gene for mitochondrial RNA, complete sequence

19.>JN400573.1 Cebus capucinus isolate CR_M003 12S ribosomal RNA gene, partial sequence; mitochondrial

20.>JN400572.1 Cebus capucinus isolate CR_cc56 12S ribosomal RNA gene, partial sequence; mitochondrial

21.>JN400570.1 Cebus capucinus isolate CR_cc02 12S ribosomal RNA gene, partial sequence; mitochondrial

22.>JN400569.1 Cebus capucinus isolate CR_A007 12S ribosomal RNA gene, partial sequence; mitochondrial

23.>AF069964.1 Alouatta palliata 12S ribosomal RNA gene, mitochondrial gene for mitochondrial RNA, complete sequence

24.>JN400591.1 Saimiri oerstedii isolate CR_CRSQ 12S ribosomal RNA gene, partial sequence; mitochondrial

25.>HQ644337.1 Saimiri oerstedii oerstedii mitochondrion, complete genome

26.>HG962416.1 Sciurus variegatoides mitochondrial partial 12S rRNA gene, specimen voucher 449883-Smithsonian National Museum of Natural History-USA

27.>HG962399.1 Sciurus granatensis mitochondrial partial 12S rRNA gene, specimen voucher 449881-Smithsonian National Museum of Natural History-USA

28.>JN393212.1 Sciurus carolinensis 12S ribosomal RNA gene, partial sequence

29.>HG962384.1 Sciurus alleni mitochondrial partial 12S rRNA gene, specimen voucher 1898.5.6.3-Natural History Museum-London-UK

30.>HG962381.1 Sciurus aberti mitochondrial partial 12S rRNA gene, specimen voucher 1938.4.1.75-Natural History Museum-London-UK

31.>HG962387.1 Sciurus colliaei mitochondrial partial 12S rRNA gene, specimen voucher 1893.2.5.10-Natural History Museum-London-UK

32.>HG962389.1 Sciurus deppei mitochondrial partial 12S rRNA gene, specimen voucher 1966.2371-Natural History Museum-London-UK

33.>HG962402.1 Sciurus igniventris mitochondrial partial 12S rRNA gene, specimen voucher 1928.7.21.68-Natural History Museum-London-UK

34.>D50287.1 Sciurus vulgaris mitochondrial 12S ribosomal RNA

35.>JF693856.1 Oecomys trinitatis voucher MUSM13320 12S ribosomal RNA gene, partial sequence; mitochondrial

36.>JF693852.1 Oecomys bicolor voucher AMNH272674 12S ribosomal RNA gene, partial sequence; mitochondrial

37.>DQ179662.1 Tylomys nudicaudus isolate TK41551 12S ribosomal RNA gene, partial sequence; mitochondrial

38.>KX786344.1 Felis catus voucher N22b 12S ribosomal RNA gene, partial sequence; mitochondrial

39.>AY495492.1 Myotis albescens 12S ribosomal RNA, tRNA-Val, and 16S ribosomal RNA genes, complete sequence; mitochondrial

40.>AY495503.1 Myotis keaysi 12S ribosomal RNA, tRNA-Val, and 16S ribosomal RNA genes, complete sequence; mitochondrial

41.>AF326099.1 Myotis nigricans 12S ribosomal RNA gene, complete sequence; tRNA-Val gene, complete sequence; and 16S ribosomal RNA gene, complete sequence; mitochondrial genes for mitochondrial products

42.>AF263236.1 Myotis riparius 12S ribosomal RNA gene, complete sequence; tRNA-Val gene, complete sequence; and 16S ribosomal RNA gene, complete sequence; mitochondrial genes for mitochondrial products

43.>AY495464.1 Eptesicus brasiliensis 12S ribosomal RNA, tRNA-Val, and 16S ribosomal RNA genes, complete sequence; mitochondrial

44.>AF326092.1 Eptesicus fuscus 12S ribosomal RNA gene, complete sequence; tRNA-Val gene, complete sequence; and 16S ribosomal RNA gene, complete sequence; mitochondrial genes for mitochondrial products

45.>L21886.1 Potos flavus mitochondrial 12S ribosomal RNA (12S rRNA) gene fragment

46.>U78344.1 Potos flavus 12S ribosomal RNA gene, partial sequence, tRNA Val gene, complete sequence and 16S ribosomal RNA gene, partial sequence, mitochondrial genes encoding mitochondrial RNAs

47.>L21888.1 Nasua nasua 12S ribosomal RNA gene, partial sequence; mitochondrial

48.>U02585.1 Procyon lotor mitochondrion 12S rRNA gene, partial sequence

49.>AB462203.1 Procyon lotor mitochondrial gene for 12S ribosomal RNA, complete sequence, specimen_voucher: personal:Tomoharu Tokutomi:NDMC-PL-OSK18160

50.>S80953.1 12S rRNA Procyon lotor=raccoons, Mitochondrial, 353 nt

51.>U12852.1 Procyon lotor mitochondrion 12S ribosomal RNA gene, partial sequence

52.>AF038014.1 Caluromys philander 12S ribosomal RNA and tRNA-Val genes, mitochondrial genes for mitochondrial products, complete sequence

53.>AF166345.1 Caluromys philander 16S ribosomal RNA gene, complete sequence; mitochondrial gene for mitochondrial product

54.>KJ868104.1 Caluromys lanatus voucher MVZ153276 mitochondrion, partial genome

55.>AJ628374.1 Didelphis marsupialis mitochondrial partial 12S rRNA gene

56.>AJ628373.1 Didelphis albiventris mitochondrial partial 12S rRNA gene

57.>AY012091.1 Didelphis virginiana 12S ribosomal RNA gene, partial sequence; and tRNA-Val gene, complete sequence; mitochondrial

58.>AF128231.1 Marmosa robinsoni 12S ribosomal RNA gene, mitochondrial gene for mitochondrial RNA, partial sequence

59.>AJ628376.1 Marmosa lepida mitochondrial partial 12S rRNA gene

60.>AJ628377.1 Marmosa mexicana mitochondrial partial 12S rRNA gene

61.>AJ628378.1 Marmosa murina mitochondrial partial 12S rRNA gene

62.>AJ628383.1 Metachirus nudicaudatus mitochondrial partial 12S rRNA gene

63.>AF128230.1 Metachirus nudicaudatus 12S ribosomal RNA gene, mitochondrial gene for mitochondrial RNA, partial sequence

64.>AF128228.1 Philander opossum 12S ribosomal RNA gene, mitochondrial gene for mitochondrial RNA, partial sequence

65.>AJ628387.1 Philander opossum mitochondrial partial 12S rRNA gene

66.>AF128234.1 Marmosops dorothea 12S ribosomal RNA gene, mitochondrial gene for mitochondrial RNA, partial sequence

67.>AJ628382.1 Marmosops pinheiroi mitochondrial partial 12S rRNA gene

68.>AJ628381.1 Marmosops parvidens mitochondrial partial 12S rRNA gene

69.>AJ628380.1 Marmosops noctivagus mitochondrial partial 12S rRNA gene

70.>AJ628379.1 Marmosops impavidus mitochondrial partial 12S rRNA gene

71.>EU851901.1 Mus musculus isolate Mus2 12S ribosomal RNA gene, partial sequence; mitochondrial

72.>LC062084.1 Mus musculus mitochondrion DNA, including 12S rRNA, tRNA-Val, 16S rRNA, tRNA-Leu genes, complete sequence, sequence_id: (92..2715)_17

73>EU851900.1 Mus musculus isolate Mus1 12S ribosomal RNA gene, partial sequence; mitochondrial

74.>LC062076.1 Mus musculus mitochondrion DNA, including 12S rRNA, tRNA-Val, 16S rRNA, tRNA-Leu genes, complete sequence, sequence_id: (92..2715)_9

75.>AF520695.1 Coendou bicolor isolate K5 12S ribosomal RNA gene, partial sequence

76.>AY093665.1 Coendou prehensilis 12S ribosomal RNA gene, partial sequence; mitochondrial gene for mitochondrial product

77.>AJ389549.1 Coendou melanurus mitochondrial 12S rRNA gene

78.>KM224250.1 Canis lupus isolate FRT22 12S ribosomal RNA gene, partial sequence; mitochondrial

79.>KM224247.1 Canis lupus familiaris isolate FRT19 12S ribosomal RNA gene, partial sequence; mitochondrial

80.>AY722397.1 Canis lupus 12S ribosomal RNA gene, partial sequence; mitochondrial

81.>Y08507.1 Canis familiaris mitochondrial 12S rRNA gene

82.>EU256476.1 Canis lupus familiaris isolate Xiasi10 D-loop, tRNA-Phe and 12S ribosomal RNA genes, complete sequence; mitochondrial

83.>KM224232.1 Bos taurus isolate FRT4 12S ribosomal RNA gene, partial sequence; mitochondrial

84.>NR_137294.1 Homo sapiens mitochondrially encoded 12S ribosomal RNA (RNR1), ribosomal RNA

85.>KM224289.1 Homo sapiens isolate FRT65 12S ribosomal RNA gene, partial sequence; mitochondrial

86.>JN034109.1 Homo sapiens isolate C960A 12S ribosomal RNA gene, partial sequence; mitochondrial

87.>DQ860680.1 Homo sapiens isolate VN-97 12S ribosomal RNA gene, partial sequence; mitochondrial

88.>KF450991.1 Homo sapiens isolate HGDP00351 mitochondrion, complete genome

89.>MG660548.1 Homo sapiens isolate Kum1615 haplogroup D5c1a mitochondrion, complete genome

90.>KJ493319.1 Mabuya sp. 1 NRPS-2014 voucher EG 027 12S ribosomal RNA gene, partial sequence; mitochondrial

91.>KJ493318.1 Mabuya sp. NRPS-2014 voucher EG-019 12S ribosomal RNA gene, partial sequence; mitochondrial

92.>DQ239210.1 Mabuya ficta voucher MBS 001 12S ribosomal RNA gene, partial sequence; mitochondrial

93.>EU477265.1 Mabuya mabouya isolate MabB 12S ribosomal RNA gene, partial sequence; mitochondrial

94.>JN227582.1 Mabuya dominicana voucher SBH268001 12S ribosomal RNA gene, partial sequence; mitochondrial

95.>KF017643.1 Lepidoblepharis xanthostigma voucher SBH:267845 12S ribosomal RNA gene, partial sequence; mitochondrial

96.>GU139948.1 Lepidoblepharis xanthostigma isolate 110G 12S ribosomal RNA gene, partial sequence; mitochondrial

97.>KF017642.1 Sphaerodactylus sp. 2 SBH-2013 voucher James R. McCranie:FN256682 12S ribosomal RNA gene, partial sequence; mitochondrial

98.>KF017640.1 Sphaerodactylus millepunctatus voucher FMNH:282795 12S ribosomal RNA gene, partial sequence; mitochondrial

99.>KF017638.1 Sphaerodactylus rosaurae voucher FMNH:282674 12S ribosomal RNA gene, partial sequence; mitochondrial

100.>KF017637.1 Sphaerodactylus elegans voucher SBH:172243 12S ribosomal RNA gene, partial sequence; mitochondrial

101.>KF258152.1 Lepidodactylus yami voucher 2011RTW05 12S ribosomal RNA gene, partial sequence; mitochondrial

102.>DQ852704.1 Lepidodactylus lugubris 12S ribosomal RNA gene, complete sequence; mitochondrial

103.>XM_018076558.1 PREDICTED: Manacus vitellinus Era like 12S mitochondrial rRNA chaperone 1 (ERAL1), mRNA

104.>AF447249.1 Piranga olivacea 12S ribosomal RNA gene, partial sequence; mitochondrial gene for mitochondrial product

105.>AF447250.1 Piranga rubra 12S ribosomal RNA gene, partial sequence; mitochondrial gene for mitochondrial product

106.>KM078808.1 Thraupis sayaca mitochondrion, partial genome

107.>EU154515.1 Turdus grayi voucher AMNH-GFB 1036 12S ribosomal RNA gene, partial sequence; mitochondrial

108.>U83765.2 Sayornis phoebe tRNA-Phe gene, partial sequence; 12S ribosomal RNA gene, complete sequence; and tRNA-Val gene, partial sequence; mitochondrial genes for mitochondrial products

109.>AF173600.1 Tyrannus tyrannus 12S ribosomal RNA gene, partial sequence; tRNA-Val gene, complete sequence; 16S ribosomal RNA gene, complete sequence; and tRNA-Leu gene, partial sequence; mitochondrial genes for mitochondrial products

110.>AY596278.1 Cnemotriccus fuscatus mitochondrion, complete genome

111.>KJ742591.1 Mionectes oleagineus voucher MJM2008 mitochondrion, complete genome

112.>MN013407.1 Gallus gallus gallus voucher whole blood mitochondrion, complete genome

113.>AJ849444.2 Gallus gallus mitochondrial partial 12S rRNA gene

114.>KP168712.1 Columba livia mitochondrion, complete genome

115.>AF433921.1 Dasyprocta punctata isolate NK14152 12S ribosomal RNA gene, partial sequence; mitochondrial gene for mitochondrial product

116.>AF520668.1 Hoplomys gymnurus isolate AK9671 12S ribosomal RNA gene, partial sequence

117.>AF422862.1 Hoplomys gymnurus clone MVZ 162309 12S ribosomal RNA gene, partial sequence; mitochondrial gene for mitochondrial product

118.>HG962379.1 Microsciurus alfari mitochondrial partial 12S rRNA gene, specimen voucher 575653-Smithsonian National Museum of Natural History-USA

119.>AY227549.1 Microsciurus flaviventer sabanillae isolate Mfl5 12S ribosomal RNA gene, partial sequence; mitochondrial gene for mitochondrial product

120.>EF156776.1 Heteromys australis LSUMZ 25452 12S ribosomal RNA gene, partial sequence; mitochondrial

121.>EF156777.1 Heteromys desmarestianus LSUMZ 36300 12S ribosomal RNA gene, partial sequence; mitochondrial

122.>U93053.1 Molossus sinaloae 12S ribosomal RNA gene, partial sequence, and tRNA-Val gene, complete sequence, mitochondrial genes encoding mitochondrial RNAs

123.>AF263215.1 Molossus molossus 12S ribosomal RNA gene, complete sequence; tRNA-Val gene, complete sequence; and 16S ribosomal RNA gene, complete sequence;

124.>MN953621.1 Oryctolagus cuniculus mitochondrion, complete genome

125.>AY012150.1 Leopardus pardalis 12S ribosomal RNA gene, partial sequence; and tRNA-Val gene, complete sequence; mitochondrial

126.>NC_028318.1 Leopardus wiedii isolate LWI mitochondrion, complete genome

127.>NC_028317.1 Leopardus tigrinus isolate LTI_CA mitochondrion, complete genome

128.>U78348.1 Urocyon cinereoargenteus 12S ribosomal RNA gene, partial sequence, tRNA Val gene, complete sequence and 16S ribosomal RNA gene, partial sequence, mitochondrial genes encoding mitochondrial RNAs

129.>JN393213.1 Urocyon cinereoargenteus 12S ribosomal RNA gene, partial sequence

130.>AM711898.1 Spilogale putorius complete mitochondrial genome

131.>KF317939.1 Odocoileus virginianus voucher 8291T_12S 12S ribosomal RNA gene, partial sequence; mitochondrial

132.>JN315624.1 Odocoileus virginianus 12S ribosomal RNA gene, partial sequence; tRNA-Val gene, complete sequence; and 16S ribosomal RNA gene, partial sequence; mitochondrial

133>MT048590.1 Sus scrofa isolate N75_H1259 small subunit ribosomal RNA gene, partial sequence; mitochondrial

134.>KX381445.1 Rattus rattus isolate M1358 12S ribosomal RNA gene, partial sequence; mitochondrial

135.>HM563837.1 Incilius luetkenii voucher UTA:A-50877 tRNA-Phe and 12S ribosomal RNA genes, partial sequence; mitochondrial

136.>KY202815.1 Rhinella icterica voucher CFBHT3640 12S ribosomal RNA gene, partial sequence; mitochondrial
